# Low Base-Substitution Mutation Rate but High Rate of Slippage Mutations in the Sequence Repeat-Rich Genome of *Dictyostelium discoideum*

**DOI:** 10.1534/g3.120.401578

**Published:** 2020-07-30

**Authors:** Sibel Kucukyildirim, Megan Behringer, Way Sung, Debra A. Brock, Thomas G. Doak, Hatice Mergen, David C. Queller, Joan E. Strassmann, Michael Lynch

**Affiliations:** * Department of Biology, Indiana University, Bloomington, IN 47405; † Department of Biology, Hacettepe University, Ankara 06800 TURKEY; ‡ Biodesign Center for Mechanisms of Evolution, Arizona State University, Tempe, AZ; § Department of Bioinformatics and Genomics, University of North Carolina, Charlotte, NC, 28223; ** Department of Biology, Washington University, St. Louis, MO 63130; †† National Center for Genome Analysis Support, Indiana University, Bloomington, IN 47405

**Keywords:** *Dictyostelium discoideum*, drift-barrier hypothesis, insertion-deletion bias, mutation, simple sequence repeats

## Abstract

We describe the rate and spectrum of spontaneous mutations for the social amoeba *Dictyostelium discoideum*, a key model organism in molecular, cellular, evolutionary and developmental biology. Whole-genome sequencing of 37 mutation accumulation lines of *D. discoideum* after an average of 1,500 cell divisions yields a base-substitution mutation rate of 2.47 × 10^−11^ per site per generation, substantially lower than that of most eukaryotic and prokaryotic organisms, and of the same order of magnitude as in the ciliates *Paramecium tetraurelia* and *Tetrahymena thermophila*. Known for its high genomic AT content and abundance of simple sequence repeats, we observe that base-substitution mutations in *D. discoideum* are highly A/T biased. This bias likely contributes both to the high genomic AT content and to the formation of simple sequence repeats in the AT-rich genome of *Dictyostelium discoideum*. In contrast to the situation in other surveyed unicellular eukaryotes, indel rates far exceed the base-substitution mutation rate in this organism with a high proportion of 3n indels, particularly in regions without simple sequence repeats. Like ciliates, *D. discoideum* has a large effective population size, reducing the power of random genetic drift, magnifying the effect of selection on replication fidelity, in principle allowing *D. discoideum* to evolve an extremely low base-substitution mutation rate.

Mutation, the ultimate source of heritable variation, may alter DNA sequence (substitutions), DNA length (insertions and deletions), and chromosome architecture (*e.g.*, duplications/translocations). Studying the mutation process is critical to understanding genetic variation within and among species and helps us to estimate the constraints on rates of evolution. Because mutations are rare events, detecting spontaneous mutations was difficult prior to the era of whole-genome sequencing. Mutation-accumulation (MA) experiments, which maintain multiple lineages at very small effective population sizes for many generations, accumulate spontaneous mutations in an effectively neutral fashion (Halligan and Keightley 2009). Whole-genome sequencing of MA lines, which allows us to determine the genome-wide rate and spectrum of spontaneous mutations, has been used to examine spontaneous mutations in a number of eukaryotic species, including *Arabidopsis thaliana*, *Caenorhabditis elegans*, *Saccharomyces cerevisiae*, *Schizosaccharomyces pombe*, *Chlamydomonas reinhardtii*, *Daphnia pulex*, *Drosophila melanogaster*, *Paramecium tetraurelia*, and *Tetrahymena thermophila* (Keightley *et al.* 2009; Farlow *et al.* 2015; Ness *et al.* 2015; Behringer and Hall 2016a; Lynch *et al.* 2016; Long *et al.* 2018b).

We performed an MA experiment using the social amoeba *Dictyostelium discoideum*, an important model organism in molecular, cellular, evolutionary and developmental biology (Kessin 2001; Williams 2010). The haploid nuclear genome of *D. discoideum* is 34 Mb and contains six chromosomes. The genome is very AT-rich: 77.6% overall and 72.5% in coding regions. Microbial eukaryotes often have unusual life histories and unique genome features (Mcgrath and Katz 2004). In *D. discoideum* these features include a high AT content (Eichinger *et al.* 2005) which contributes to the formation of simple sequence repeats (SSRs) (Tian *et al.* 2011), genetic loci where one or a few bases (∼10) are tandemly repeated a varying number of times. Unlike other eukaryotes where SSRs typically only account for 2% of the genome and consist mainly of mono- and dinucleotide repeats, SSRs make up over 14.3% of the *D. discoideum* genome (Srivastava *et al.* 2019) and are biased toward repeat units of 3 and 6 bases (Eichinger *et al.* 2005). Moreover, these SSRs are found in over 2,000 (>16.3%) *D. discoideum* protein-coding genes (Eichinger *et al.* 2005), comprising ∼3.5% of the coding bases in the total genome.

The *D. discoideum* vegetative MA lines used in this study were maintained in two separate locations, the Queller-Strassmann (QS) Lab in St. Louis, MO and the Lynch (L) Lab in Bloomington, IN. We confirmed, with a much larger sample size, the previous finding (Saxer *et al.* 2012; Hall *et al.* 2013) that the *D. discoideum* base-substitution mutation rate is one of the lowest observed to date and comparable to that in other unicellular eukaryotes (Sung *et al.* 2012a; Sung *et al.* 2012b; Ness *et al.* 2015; Long *et al.* 2016; Krasovec *et al.* 2017). In addition, our results allow us to determine the genome-wide spectrum and distribution of spontaneous mutations, as well as the high rate of slippage mutations in an SSR-rich genome, even in protein-coding regions.

## Materials And Methods

### Mutation accumulation, DNA extraction and sequencing

Two independent sets of *Dictyostelium discoideum* AX-4 haploid MA lines were used in this analysis, cultured in separate labs (Lynch (L) and Queller-Strassmann (QS)) at separate institutions. These two sets experienced the same standard MA protocols: independent *D. discoideum* AX4 MA lines (60 L lines and 100 QS lines) were initiated from a single colony and grown on Petri dishes containing SM/5 medium (Mcconnell *et al.* 2007); every second day a haphazardly chosen single colony from each MA line was transferred by streaking to a new plate, ensuring that each line regularly passed through a single-cell bottleneck (Mcconnell *et al.* 2007). As described in Saxer *et al.* (2012), we estimated the number of generations during each bottleneck interval. This yielded an estimate of ∼14.2 generations, and growth rate on plates did not change notably during the course of the experiment. Multiplying this number by the transfer number yields the total generations experienced by each line. The bottlenecking procedure used for this experiment ensures that mutations accumulate in an effectively neutral fashion (Kibota and Lynch 1996). During our MA experiment, we observed no fruiting bodies, indicating that all cells remained in the vegetative single-cell stage. Every 10 transfers, spores were collected and stored at –80°.

The L set of MA lines was carried for ∼2000 generations with 60 independent lineages, all derived from a single ancestral colony. We sequenced 20 of the L MA lines so that approximately 40,000 generations of mutation accumulation were acquired. With this data set, we were able to determine the natural mutation spectrum of *D. discoideum*.

The QS set of MA lines were derived from a previous study, involving a large-scale MA experiment using the same strain of *D. discoideum* carried for ∼1000 generations. However, only three of these 100 lines were sequenced, and only one mutation was identified (Saxer *et al.* 2012). In order to supplement our results and confirm the repeatability of MA experiments across laboratories, we again applied high-throughput sequencing to 20 of the 100 QS MA lines from this prior experiment.

To extract DNA from the QS MA lines (Saxer *et al.* 2012) for whole genome sequencing, 1-2x10^8^ amoeba cells in liquid culture were collected by centrifugation at 300 g for 3 min at 4°, washed with ice-cold starvation buffer (2.25 g KH_2_PO_4_ and 0.67 g K_2_HPO_4_), and centrifuged again. Genomic DNA was then extracted using the Blood & Cell Culture Maxi kit (Qiagen) according to the manufacturer`s protocol. Similarly, DNA for the L MA lines was extracted from 20 *D. discoideum* MA lines using the Wizard extraction kit (Promega, Madison, Wisconsin, USA). DNA libraries for Illumina HiSeq 2500 sequencing were constructed using the Nextera DNA Sample Preparation kit (Illumina, San Diego, CA). Paired-end 150 nt read sequencing of MA lines was performed by the Hubbard Center for Genome Studies, University of New Hampshire.

### Mutation identification and analyses

Adaptors of paired-end reads were removed with Trimmomatic 0.32 (Bolger *et al.* 2014); trimmed reads for each MA line were individually mapped to the reference genome (NCBI accession numbers: NC_007087-NC_007092) using the BWA alignment algorithm, version 0.7.12 (Li and Durbin 2009). Due to the highly repetitive nature of the genome, centromeres and telomeres could not be mapped properly by short-read alignment algorithms, thus these regions were excluded from the final analyzed sites. The output was sorted and indexed with SAMTOOLS (Li *et al.* 2009); we also applied duplicate-read removal using picardtools-2.5.0 (Mckenna *Et Al*. 2010). To ensure highly accurate calling of mutations we set a cutoff of 20x sequencing coverage to be included in the final analysis. As such, three MA lines (QS34, QS89 and L18) were excluded from further analyses because of significantly lower sequencing coverage than other lines (<20x) (Supplemental Table 1). This resulted in an average of 36.7x coverage across the remaining 37 MA lines.

Base-substitution mutations and small (1-30bps) insertion-deletions for each MA line were identified using the HaplotypeCaller tool in GATK_3.6 (Depristo *et al.* 2011) with standard hard filtering parameters described by GATK Best Practices Recommendations (except that we set the MQ ≥ 60 for both variant and non-variant sites). In order to call a variant, a minimum of ten reads was needed. In addition to a consensus approach, because short-read mapping algorithms have difficulties in mapping indel events >10bp, we also used NOVOALIGN (available at www.novocraft.com) and PINDEL (Ye *et al.* 2009) algorithms to ensure that alignment errors are not responsible for false-positive variant calls. We included only the indels called by all the three algorithms in our analysis. Repeat regions larger than the library insert size could not be resolved. We also used in-house perl scripts to detect variants located in SSRs (https://cci-git.uncc.edu/wsung/ssrsearch). All mutation sites were confirmed by visual examination using Integrated Genomics Viewer (IGV) (Thorvaldsdottir *et al.* 2013). Identified base substitutions and indels were annotated using SnpEff (Cingolani *et al.* 2012).

### Mutation-rate calculations

The base-substitution mutation rate ($\mu_{bs}$; per nucleotide site per cell division) was calculated for each line as $\mu_{bs}\;=\;\frac{m}{\left(nT\right)}$, where *m* is the number of observed base-substitution mutations, *n* is the number of sites analyzed in the line, and *T* is the number of generations that occurred in the line. Bootstrapped confidence intervals describing the variation in *n* and *T* was estimated using the boot package for R with the BCa adjustment for 1000 bootstrap replicates. The expected probability of occurrence of multinucleotide mutations (MNM) within the window size 50 nucleotides in the genome of *D. discoideum* MA lines was calculated as in Senra *et al.* (2018).

The GC content at mutation equilibrium was calculated as (Lynch 2007): $\frac{\;\mu {\;}_{A/T\to G/C}}{\mu {\;}_{G/C\to A/T}\;+\;\mu {\;}_{A/T\to G/C}}$, where *μ_A/T→G/C_* is the number of A/T mutations at A:T sites resulting in an A/T → G/C change (including A/T → G/C transitions and A/T → C/G transversions) divided by the product of the number of A/T sites and the number of generations; and *μ_G/C→A/T_* is the number of G/C mutations at G:C sites resulting in a G/C → A/T change (including G/C → A/T transitions and G/C → T/A transversions) divided by the product of the number G/C sites and the number of generations (Supplemental Table 1). Mutation bias in the G/C direction was calculated by $\frac{\;\mu {\;}_{A/T\to G/C}}{\mu {\;}_{G/C\to A/T}\;}$. We used R V3.I.0 (R Development Core Team 2014) for all statistical tests. 95% Poisson confidence intervals were calculated using the Poisson test in R.

### Data availability

Workflow deposited at the GitHub repository (https://github.com/sibelkucukyildirim/Dicty) and raw sequences are available at the Sequence Read Archive at NCBI (Bioproject No.: PRJNA615815). Supplemental material available at figshare: https://doi.org/10.25387/g3.12732311.

## Results

### Base-substitution mutations

We calculated the base-substitution mutation rate for each of the MA lines by dividing the number of determined base-substitutions by the number of nucleotide sites analyzed and the estimated number of generations that occurred over the course of the MA experiment. Across the 37 MA lines (with an average 81.3% of the genome analyzed per line), we identified 37 base substitutions, yielding an overall rate of 2.47 (SE = 0.54) × 10^−11^ per site per generation (Supplemental Table 1). There was no statistical difference between MA-line specific mutation rate estimates (*P* = 0.76): the average per site per generation base-substitution mutation rate observed in the 19 *D. discoideum* L MA-lines, $\mu_{bs}$= 2.34 (SE= 0.66) × 10^−11^, and the 18 QS MA lines, $\mu_{bs}$ =2.68 (SE= 0.87) × 10^−11^.

**Figure 1:**
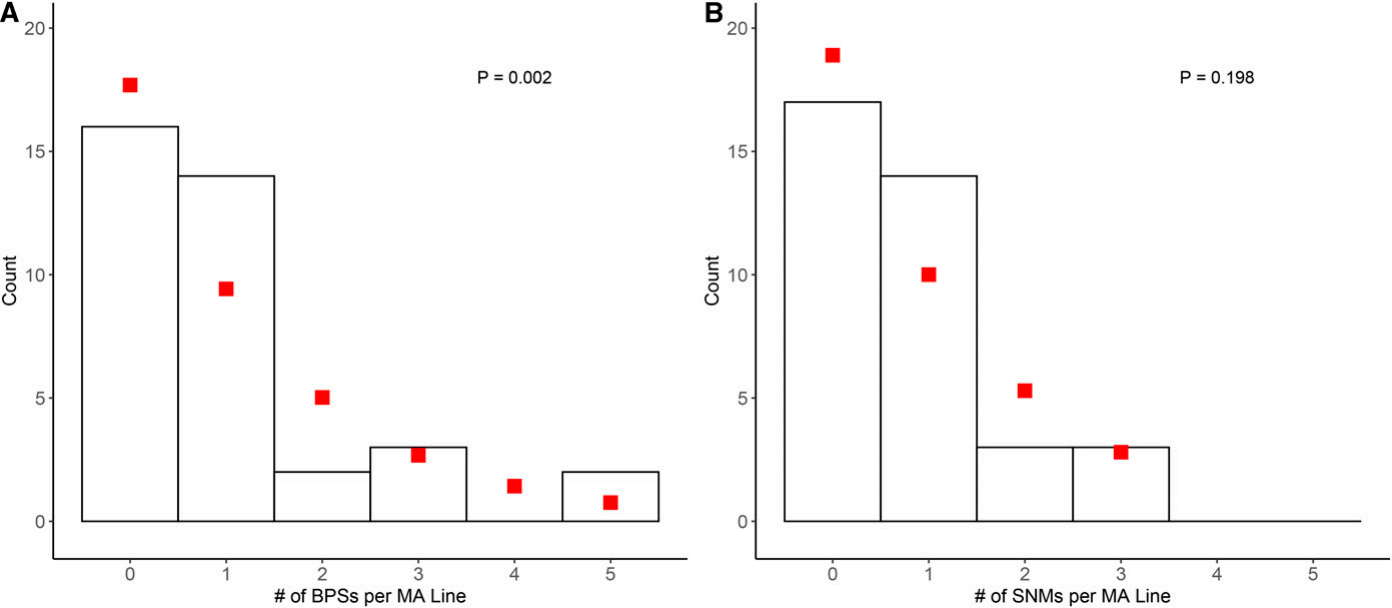
Histograms describing number of point mutations identified in each mutation accumulation line. Bars represent the number of mutation accumulation (MA) lines observed to contain a particular number of (A) total base pair substitutions (BPSs) and (B) total single nucleotide polymorphisms (SNPs) after correction for multinucleotide mutations (MNMs; BPSs and indel mutations which occur within 50 bp and are unlikely to be independent). Red squares represent the fitted expectation of counts based on a Poisson distribution and p-value describes goodness of fit.

Across the 37 MA lines, an average of 1 base substitution per line was observed (16 lines did not accumulate any base substitutions). If each base substitution is truly an independent event, then the number of base substitutions per line should fit a Poisson distribution. However, this was not the case (χ^2^ = 14.21, *P* = 0.002), suggesting that variation in number of generations and number of genomic sites covered across MA lines may lead to violation of Poisson distribution or that some MA lines accumulated multiple mutations through non-independent events (Figure 1A, Supplemental Table 1 and 2). One factor that can cause mutations to appear to accumulate in a non-Poisson fashion is multi-nucleotide mutation (MNM; defined as mutations that occur within 50 nucleotides of each other in a single MA line) (Schrider *et al.* 2011). Assuming that mutations are randomly distributed in the genome, the probability that two or more mutations arose independently within a window of 50 nucleotides is extremely low (*P* = 1.47× 10^−6^ per line on across all MA lines). Thus, MNMs are likely the result of two independent events and are instead more likely to be the result of a single mutational event or a local mutational hotspot.

MNMs have been reported in previous MA experiments with haploid unicellular eukaryotes (*S. cerevisiae* (Sharp *et al.* 2018), *S. pombe* (Behringer and Hall 2016a), *C. reinhardtii* (Ness *et al.* 2015)) and assorted marine green algae (Krasovec *et al.* 2017)). They are a possible result of double-stranded break repair by non-homologous end joining (NHEJ) (Rodgers and Mcvey 2016), as homologous recombination in haploids is limited to when a daughter chromatid is available (S phase and G2 phases of the cell cycle (Heyer *et al.* 2010)). Here, we found that 4 out of the 19 MA lines maintained in the Lynch Lab had acquired MNMs, accounting for 8 out of all 37 base-substitutions (Supplemental Table 2). As such, counting MNMs as multiple mutational events instead of a single mutational event may have contributed to the poor fit of our data to a Poisson distribution. Taking this possibility into account, we re-calculated the number of base substitutions per line and found the corrected number of mutational events among lines to be more consistent with a Poisson distribution (χ^2^ = 3.23, *P* = 0.19) (Figure 1B).

### Mutation spectrum

In eukaryotic species assayed to date, two patterns appear to be near universal— a G/C→A/T mutation bias, and a transition/transversion (T_s_/T_v_) base-substitution ratio greater than the random expectation of 0.5 (Petrov and Hartl 1999; Hershberg and Petrov 2010; Long *et al.* 2018b). Consistent with these eukaryotic species, we also observe a G/C→A/T mutation bias in *D. discoideum*. Specifically, we calculate an A/T→G/C mutation rate of 0.44 ×10^−11^ per site per generation (95% CI: 0.14 to 1.03 ×10^−11^; 4 transitions, 1 transversion) and a G/C→A/T mutation rate of 4.36 ×10^−11^ per site per generation (95% CI: 2.49 to 7.07 ×10^−11^; 7 transitions, 9 transversions) (Supplemental Table 1). However, *D. discoideum* deviates from other species as mutations are biased toward transversions with a Ts/Tv ratio of 0.42 (11 transitions *vs.* 26 transversions). Given the conditional A/T↔G/C mutation rates, the expected GC content at mutation equilibrium is 9.2%, significantly lower than the actual genome GC content of 22.5%. This is consistent with prior MA experiments where the expected GC content is lower than the observed GC content, and further evidence that GC content is maintained by other evolutionary forces such as natural selection (Zhu *et al.* 2014; Farlow *et al.* 2015; Behringer and Hall 2016a; Long *et al.* 2018b). Further, there was also no significant difference in the types of base-substitutions (mutation spectrum) that accumulated in the two independent sets of MA lines ( χ^2^ = 3.73, df = 5, *P* = 0.59) (Supplemental Table 1 and Supplemental Figure 1).

### Small insertions and deletions

We found a total of 74 small insertions and deletions (1-30 bps in length) across the 37 MA lines, yielding an indel-mutation rate of 4.93 (SE= 0.86) × 10^−11^ per nucleotide site per generation. The total indel rate is significantly different between the two independent MA sets (6.70×10^−11^ in L lines and 1.94×10^−11^ in QS lines) (Supplemental Tables 1 and 3) (*t*-test, *P* = 0.0059). This difference remains when independently comparing insertion and deletion rates between the two groups of MA lines (*t*-test; insertions: *P* = 0.084, deletions: *P* = 0.006) as indel rate differences within an order of magnitude are not uncommon within a species (Behringer and Hall 2016b).

**Figure 2:**
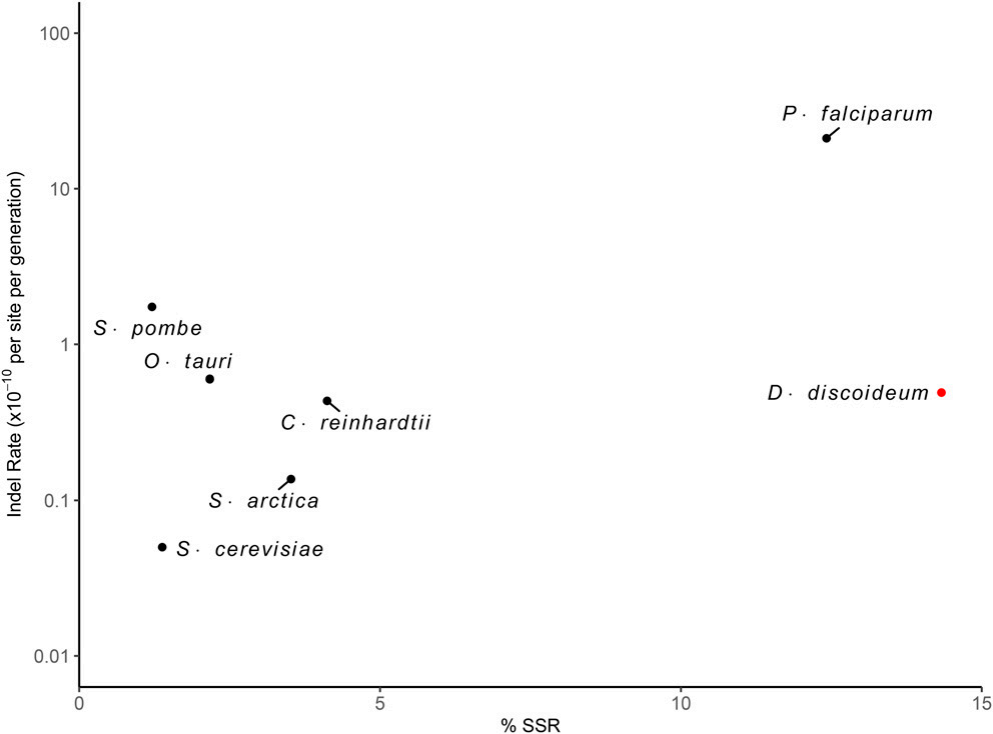
The relationship between the indel mutation rate and the abundance of simple sequence repeats within the genome (%). Red data points highlight the observed indel rate of *Dictyostelium discoideum* from this study with respect to percent abundance of simple sequence repeats (SSRs; Srivastava *et al.* 2019). Black data points correspond to the following additional unicellular eukaryote species for reference: *Chlamydomonas reinhardtii* (Sung *et al.* 2012a); *Ostreococcus tauri* (Krasovec *et al.* 2017); *Schizosaccharomyces pombe* (Behringer and Hall 2016a); *Saccharomyces cerevisiae* (Zhu *et al.* 2014); *Plasmodium falciparum* (Hamilton *et al.* 2017); *Sphaeroforma arctica* (Long *et al.* 2018b).

These rates of small insertions and deletions reveal a source of mutational bias in *D. discoideum*, as the indel rate is 2x higher than the base-substitution rate and the deletion rate is 4.5x higher than the insertion rate. Recent studies suggest such a bias in indel rate to base-substitution mutation rate is typical, as biases ranging between 0.14 and 8.6 have been observed (Sung *et al.* 2012a; Behringer and Hall 2016a; Hamilton *et al.* 2017; Krasovec *et al.* 2017; Long *et al.* 2018b) (Figure 2). However, in contrast to our results, some unicellular eukaryotes are reported to exhibit insertion biases (Sung *et al.* 2012a; Sung *et al.* 2012b; Farlow *et al.* 2015; Behringer and Hall 2016a) although deletion biases have been previously observed in prokaryotes (Sung *et al.* 2016).

### Functional effects of single-nucleotide mutations and small indels

Using the annotation of the *D. discoideum* genome (Eichinger *et al.* 2005), we identified the functional context of each base substitution and indel event (Table 1, Supplemental Table 3 and 4). Across the 37 lines, 25 of the 37 (67.6%) substitutions are in protein-coding regions, while 12 are in non-coding sites. Given the codon usage and transition/transversion ratio in *D. discoideum*, the expected ratio of nonsynonymous to synonymous mutations is 3.32 if selection was ineffective, which is not significantly different from the observed ratio of 5.25 (21/4) (χ^2^ = 0.88, df = 1 *P* = 0.35). Therefore, we did not find an evidence indicating that selection biased this experiment. Our analysis revealed that 46 of the 74 (62.2%) small insertion/deletion events are in protein-coding regions and the remaining 28 are in noncoding regions. These indels were randomly distributed across the genome, and the observed events in protein-coding regions are not different than expectations (observed = 46, expected = 46). Also, around 20% of all protein-coding mutations occurred in SSRs (Supplemental Table 3 and 4).

**Table 1:** Mutation patterns in *D. discoideum* mutation accumulation lines

	Experiment I (Queller-Strassmann)	Experiment II (Lynch)	Overall
No. of sites surveyed	31113614	24222775	55336389
No. of base-substitutions	15	22	37
No. of indels	11	63	74
Transitions/Transversions	0.36	0.46	0.44
No. of synonymous substitutions	3	1	4
No. of nonsynonymous substitutions	7	14	21
No. of noncoding substitutions	5	7	12
No. of base substitutions in SSRs	6	6	12
No. of indels in SSRs	6	18	24
No. of indels in coding regions	3	46	49
No. of indels in noncoding regions	8	17	25
Base-substitution mutation rate (× 10^−11^)	2.68	2.34	2.47
Insertion-deletion rate (× 10^−11^)	1.94	6.70	4.93

Focusing further on SSRs, single nucleotide base-substitutions occurred in SSR regions significantly more often than expected by chance (L MA-lines: 27.3%, Q MA-lines: 40%; *P* = 0.0008). Additionally, 32.4% of indel events (L MA-lines: 28.6%, Q MA-lines: 54.5%; *P* < 0.0001) occurred in SSRs, primarily in homopolymeric A:T runs, as SSRs are commonly known to be mutational hotspots for insertion-deletion events (Toth *et al.* 2000)(Supplemental Table 3). Despite *D. discoideum*’s SSR-rich genome, we were unable to examine the relationship between length of SSRs (number of repeats) and the number of indels due to limited statistical power (Supplemental Figure 2).

### Effective population size

**Figure 3:**
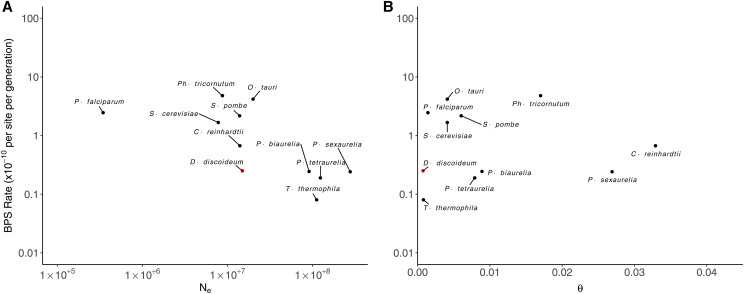
The relationships between the base-substitution mutation (BPS) rate and predictors of population-level genetic diversity. The effective population size (A) and θ (B) in unicellular eukaryotes. Red data points highlight the observed base pair substitution (BPS) rate of *Dictyostelium discoideum* from this study with respect to effective population size (*N_e_*) and nucleotide diversity (θ) (Flowers *et al.* 2010). Black data points correspond to the following additional unicellular eukaryote species for reference: *Chlamydomonas reinhardtii* (Smith and Lee 2008; Sung *et al.* 2012a; Ness *et al.* 2015); *Paramecium biaurelia*, *Paramecium sexaurelia* (Long *et al.* 2018a); *Paramecium tetraurelia* (Sung *et al.* 2012b); *Ostreococcus tauri* (Krasovec *et al.* 2017); *Plasmodium falciparum* (Lynch 2010; Hamilton *et al.* 2017); *Saccharomyces cerevisiae* (Schacherer *et al.* 2009; Zhu *et al.* 2014); *Schizosaccharomyces pombe* (Fawcett *et al.* 2014; Behringer and Hall 2016a); *Tetrahymena thermophila* (Long *et al.* 2016); *Phaeodactylum tricornutum* (Krasovec *et al.* 2019).

For genomic sites assumed to be evolving neutrally, the amount of genetic variation maintained in a population is determined by the strength of drift, measured as the effective population size (*N_e_*), and the mutation rate (μ), with the expected value of the nucleotide diversity being 2*N_e_*μ for haploids. We can therefore use our mutation-rate estimate (μ) to estimate the effective population size in *D. discoideum*, using a published measurement of the standing heterozygosity/nucleotide sites of θ=0.00076 (Flowers *et al.* 2010) with the estimated μ_bs_ (2.47 × 10^−11^) in this study: we estimate *N_e_* to be ∼1.5 × 10^7^ (with a 95% confidence interval ranging from 0.74-2.49 × 10^7^). Compared to other eukaryotic microbes, *N_e_* of *D. discoideum* is more similar to *Chlamydomonas reinhardii* (1.4 ×10^7^) (Ness *et al.* 2015) than to *Paramecium tetraurelia* (1.2 ×10^8^) (Sung *et al.* 2012b) and *Tetrahymena thermophila* (1.1 × 10^8^) (Long *et al.* 2016) (Figure 3).

## Discussion

Microbial eukaryotes are an extremely diverse group, containing many evolutionarily distant lineages, some of which have unusual life histories and genome features (Mcgrath and Katz 2004; Eichinger *et al.* 2005). Their unique features often make it difficult to design MA experiments, which require the cultivation and maintenance of lines at a small effective population size. Thus, we have been limited in our understanding of how microbial eukaryotes mutate and evolve.

Here, whole-genome sequencing of 37 mutation-accumulation lines of *D. discoideum* after ∼1,500 cell divisions yielded a base substitution mutation-rate estimate of 2.47 × 10^−11^ per site per generation. Although the mutation rate of *D. discoideum* is lower than that observed for most other eukaryotes, it is comparable to that of the ciliates *P. tetraurelia* and *T. thermophila* (Sung *et al.* 2012b; Long *et al.* 2016). Previous work has suggested that the effective population size of an organism determines the efficiency of selection to reduce mutation rates (Lynch 2011; Sung *et al.* 2012a; Lynch *et al.* 2016), and the low base-substitution mutation rate observed in *D. discoideum* is consistent with its high effective population size and low genetic diversity, although more detailed information on this matter would be helpful. In principle, the low mutation rates in the ciliates *P. tetraurelia* and *T. thermophila* may also be a function of the silent accumulation of mutations in the micronucleus during vegetative propagation, which are exposed only after periods of sexual reproduction, the relevant timescale for the operation of selection on the mutation rate (Sung *et al.* 2012b). However, *D. discoideum* has managed to evolve an extremely low base-substitution mutation rate without the latter complications, suggesting that the primary determinant of mutation rate evolution in these unicellular species may be associated with effective population size.

The effective population size of *D. discoideum* (*N_e_*= 1.5 × 10^7^) is estimated to be lower than that in the ciliates *T. thermophila* (*N_e_* = 1.1 × 10^8^) (Long *et al.* 2016) and *P. tetraurelia* (*N_e_* = 1.2 × 10^8^) (Sung *et al.* 2012b), but similar to that in *Chlamydomonas reinhardtii* (*N_e_* between 1.4 × 10^7^ and 3.1 × 10^7^) (Sung *et al.* 2012a; Ness *et al.* 2015). The low base-substitution mutation rate of *D. discoideum* cannot readily be explained by an increase in the effective genome size (target size for deleterious mutations in the genome, determined by the coding size of the genome including synonymous sites), as the effective genome size of *D. discoideum* (∼21 Mb, 62% of its total genome) is smaller than that of *P. tetraurelia* (∼56 Mb, 78% of its macronucleus’ genome). However, the expected mutation rate defined by the effective population size (drift barrier) is not deterministic, and at any point in time there can be a substantial range of variation around the drift-barrier expectation (Lynch 2011). Therefore, for such a small group of taxa, we cannot rule out the possibility that inconsistencies of mutation rates with respect to the drift barrier are simply a consequence of evolutionary stochastic variation.

### Insertion/deletion bias

Although *D. discoideum* is a well-known model organism, some of its genome features complicate computational approaches to detect mutations. The *D. discoideum* genome is AT-rich (over 77%) (Eichinger *et al.* 2005) and 14.3% of the genome consists of simple sequence repeats (Srivastava *et al.* 2019), much higher than most other sequenced organisms, which could result in a high indel rate. A general observation in a wide range of species is that indels commonly occur in repeat regions and the base-substitution mutation rate is ∼10x greater than the per nucleotide site indel rate (Sung *et al.* 2016). However, our analysis reveals that the rate of indel mutations in *D. discoideum* is also high in non-SSR regions and that its indel rate is elevated twofold relative to its base-substitution rate. An overall indel rate of 4.93 (SE= 0.86) × 10^−11^ per site per generation is unusually high compared other organisms, especially unicellular eukaryotes (Long *et al.* 2016; Sung *et al.* 2016). However, a recent study conducted with *Plasmodium falciparum*, which has similar genomic features (AT content >80%, SSR content = 12.42%) to *D. discoideum*, comparably showed an indel rate that is over ten-fold higher than its base-substitution rate (Hamilton *et al.* 2017).

Previously, a pervasive bias has been reported toward deletions in all taxonomic groups examined: archaea, bacteria, nematodes, insects, and mammals (Kuo and Ochman 2009). But a bias toward insertions relative to deletions has been observed for some eukaryotes (Denver *et al.* 2004; Lynch *et al.* 2008; Sung *et al.* 2012a; Sung *et al.* 2012b; Farlow *et al.* 2015; Behringer and Hall 2016a). Our results indicate that deletion events outnumber insertions in *D. discoideum*, consistent with a recent study in *C. elegans* (Saxena *et al.* 2019). Small deletions occurred ∼4.5× more frequently than insertions (the total size of all deletions is 545 bp while the insertions total 59 bp), resulting in a net loss of 486 bp in DNA sequence across all lines (average 13 (SE= 3.8) bp loss per line). If we assume an equilibrium genome size that is driven by mutation pressure alone, this would suggest that either the *D. discoideum* genome is shrinking or that selection is acting against deletion bias to maintain its current genome size (Petrov 2002; Gregory 2004). Considering the high proportion of SSRs in coding regions, selection may act against deletions to preserve protein function and offset the loss of DNA through frequent small deletions.

Because highly repetitive regions of the genome were excluded in our analysis, the mutation rates observed at SSRs are likely to be an underestimate of the total SSR rate. However, we still found that more than half of indels that fall in SSR regions are in coding regions, suggesting that the SSR-rich genome of *D. discoideum* imposes a significant substrate for the development of mutational load. Because SSRs in both prokaryotes and eukaryotes represent hypermutable loci, selection should operate to reduce the mutation rate on SSRs or eliminate SSRs altogether, especially if they are affecting coding sequences. *D. discoideum*, like other unicellular eukaryotes, has a population size large enough to make selection quite effective (Lynch 2010). The distribution of repeats and simple-sequence tracts in *D. discoideum* genome is non-random, suggesting that they are tolerated only in certain types of protein (Eichinger *et al.* 2005), but work by Scala *et al.* (2012) showed that the SSRs located in coding regions are highly variable in length and suggested that these SSRs primarily evolve by mutation and drift and are not strongly selected upon. Thus, the issue of why *D. discoideum* contains large numbers of SSRs in its genome may be explained by the idea that a very high AT (or GC) genome content may be driven by a mutational bias that may lead to the accumulation of random sequences with repeats (Tian *et al.* 2011).

## Conclusion

The drift-barrier hypothesis (Lynch 2010; Lynch 2011; Sung *et al.* 2012a) suggests that selection will operate to reduce the mutation rate to minimize deleterious mutations, with genetic drift presenting a barrier below which selection for further improvement will be ineffective. Under this hypothesis, organisms with a similar effective population size and genome size—and thus effect of genetic drift—should have similar mutation rates. However, those organisms can differ in their mutation spectrum, because selection can generate a similar overall mutation rate using different mechanisms to maintain relative replication fidelity. Our estimate of the base-pair mutation rate of *D. discoideu*m is similar to the previous estimates for *P. tetraurelia* (Sung *et al.* 2012b) and *T. thermophila* (Long *et al.* 2016), but it appears that in these unicellular eukaryotes, effective population sizes and genome sizes are different. The genome size of *D. discoideum* may reduce the ability of selection to minimize replication errors, which may lead to a slightly higher mutation rate compared to the two ciliates.
